# A Combination Approach in Inhibiting Type 2 Diabetes-Related Enzymes Using *Ecklonia radiata* Fucoidan and Acarbose

**DOI:** 10.3390/pharmaceutics13111979

**Published:** 2021-11-22

**Authors:** Blessing Mabate, Chantal Désirée Daub, Samkelo Malgas, Adrienne Lesley Edkins, Brett Ivan Pletschke

**Affiliations:** 1 Enzyme Science Programme (ESP), Department of Biochemistry and Microbiology, Faculty of Science, Rhodes University, Makhanda 6140, South Africa; g18M0025@campus.ru.ac.za (B.M.); g15D2439@campus.ru.ac.za (C.D.D.); 2 Department of Biochemistry, Genetics and Microbiology, University of Pretoria, Pretoria 0028, South Africa; samkelo.malgas@up.ac.za; 3 Biomedical Biotechnology Research Unit, Department of Biochemistry and Microbiology, Faculty of Science, Rhodes University, Makhanda 6140, South Africa; a.edkins@ru.ac.za

**Keywords:** acarbose, combination approach, *Ecklonia radiata*, fucoidan, type 2 diabetes management

## Abstract

Although there are chemotherapeutic efforts in place for Type 2 diabetes mellitus (T2DM), there is a need for novel strategies (including natural products) to manage T2DM. Fucoidan, a sulphated polysaccharide was extracted from *Ecklonia radiata*. The integrity of the fucoidan was confirmed by structural analysis techniques such as FT-IR, NMR and TGA. In addition, the fucoidan was chemically characterised and tested for cell toxicity. The fucoidan was investigated with regards to its potential to inhibit α-amylase and α-glucosidase. The fucoidan was not cytotoxic and inhibited α-glucosidase (IC_50_ 19 µg/mL) more strongly than the standard commercial drug acarbose (IC_50_ 332 µg/mL). However, the fucoidan lacked potency against α-amylase. On the other hand, acarbose was a more potent inhibitor of α-amylase (IC_50_ of 109 µg/mL) than α-glucosidase. Due to side effects associated with the use of acarbose, a combination approach using acarbose and fucoidan was investigated. The combination showed synergistic inhibition (>70%) of α-glucosidase compared to when the drugs were used alone. The medicinal implication of this synergism is that a regimen with a reduced acarbose dose may be used, thus minimising side effects to the patient, while achieving the desired therapeutic effect for managing T2DM.

## 1. Introduction

Type 2 diabetes mellitus (T2DM) remains one of the prominent emerging chronic diseases in humans, with about 400 million people living with diabetes worldwide [1]. T2DM is a burden to the world in mortality, morbidity and disability-adjusted life years (DALYs), which significantly steers economic pressure in medium or low-income countries [1]. Diabetes is projected to be the seventh-highest global cause of death by 2030 [2]. T2DM is characterised by hyperglycemia, leading to considerable damage to organs, nervous and cardiovascular systems [3]. The main source of blood glucose levels is carbohydrate (starch) hydrolysis by catabolic enzymes in the alimentary canal, namely α-amylase and α-glucosidase [4]. Carbohydrate digestion directly impacts the amount of glucose and sugars absorbed into the bloodstream, causing hyperglycaemia, especially in insulin-resistant individuals [5]. Therefore, slowing down carbohydrate digestion or inhibiting glucose absorption is a promising approach for treating diabetes and its complications [6].

Several strategies to manage T2DM exist, including insulin secretagogues, insulin sensitisers and acarbose, a semi-synthetic compound that inhibits carbohydrate digesting enzymes [4,7]. However, as with most chemotherapeutics, the use of acarbose is associated with various side effects, including flatulence, abdominal discomfort and diarrhoea [8]. Despite the promising success of chemotherapeutic drugs in clinical trials, side effects associated with cytotoxicity often surface. Therefore, there is a need for developing better-tolerated treatments with improved therapeutic properties. Natural bio-compounds, including marine products, are known to have therapeutic advantages over chemotherapeutics [9]. Fucoidans are among the major marine polysaccharides that have gained interest as α-amylase and α-glucosidase inhibitors [10,11]. Besides their inhibition potency on the digestive enzymes mentioned, fucoidans have demonstrated anti-diabetic potential by inhibiting dipeptidyl peptidase IV (DPPIV) [12]. Also, fucoidans have shown anti-diabetic potential in vivo where they have reduced blood sugar levels in induced hyperglycemic rats [13]. The anti-diabetic relevance of fucoidans is profound and must be further elucidated.

Fucoidans are complex anionic sulphated polysaccharides composed mainly of fucose and varying monosaccharides in their structure. Fucose residues are linked by α-(1-3), α-(1-3)-α-(1-4) or α-(1-3)-α-(1-2) linkages and sulphate groups that are attached to the O-2 and/or O-4 position of the sugar residues composing the polysaccharide backbone [4]. The biological activity of fucoidan has gained remarkable attention in biomedical studies, including T2DM alleviation efforts [10,11]. However, there is limited literature on fucoidan extracted from South African seaweeds despite the fact the country has one of the largest coastlines globally, with vast algal biodiversity [14].

Combining multiple drugs is a common approach in the biomedical and pharmaceutical sciences [15]. The rationale behind drug combinations with non-overlapping side effects is to obtain regimens with lowered adverse effects, avoiding resistance to single compounds and possibly obtaining a direct empirical synergy of the compounds against a targeted biological process [16]. Although there are so many conflicting definitions of synergy —in this context, it is defined as a combined experimental effect greater than the additive effects of individual compounds. Drug synergism has been quantified in vitro and in vivo by two widely accepted reference models: the Loewe Additivity and the Bliss Independence [17]. The Loewe model describes the additive effect of two compounds with the same mode of action, while the Bliss model describes the additive effect of two ingredients with different modes of action [17]. Among the reference models for measuring synergy, the Chou-Talalay method has become the most popular and used model [17]. The median effect equation, characteristic of the Chou-Talalay method, was derived from a unified theory of Michaelis-Menten, Hill, Henderson-Hasselbalch and Scatchard equations [18]. Despite the limitations of this reference model, it is best suited for drug combination studies involving enzymes [18].

It has been established that acarbose inhibits α-amylase significantly more than it does α-glucosidase; the inverse has been observed for most characterised fucoidans [10,19]. Therefore, drug combination studies for acarbose and fucoidan are a plausible approach for leveraging each compound’s inhibitory potential. This study sought to characterise *Ecklonia radiata* derived fucoidan and demonstrate its potency as an inhibitor of α-amylase and α-glucosidase, significant targets in T2DM therapy. Furthermore, drug combination studies using acarbose and *E. radiata* fucoidan were explored and shown to inhibit α-glucosidase to a significantly higher degree than when the two compounds were used alone.

## 2. Materials and Methods

The fucoidan was extracted from harvested *E. radiata* seaweed species. Both the fucoidan obtained from *Fucus vesiculosus* (Cat. No. F5631) and acarbose (Cat No A8980) were purchased from Sigma-Aldrich (St. Louis, MO, USA). The two amylolytic enzymes, porcine pancreatic α-amylase (Cat. No. E-PANAA) and *Saccharomyces cerevisiae* α-glucosidase (Cat. No. G5003), were purchased from Megazyme^TM^ (Bray, Ireland) and Sigma-Aldrich, respectively. The rest of the reagents used in the study were of analytical grade and purchased from Sigma-Aldrich, MERCK (Darmstadt, Germany) and Megazyme^TM^.

### 2.1. Harvesting and Preparation of Seaweeds

The brown seaweed, *E. radiata*, was harvested from the South African Indian Ocean coastline in the Eastern Cape province at the coordinates: 33°36′ 36.8424″ S; 26°53′ 23.4996″ E. The harvested seaweed was stored on ice during transportation to the laboratory. Upon arrival in the laboratory, the seaweed was washed thoroughly with distilled water, cut into small pieces and dried at 40 °C in an oven for 72 h. The dried seaweed samples were subsequently pulverised with a coffee bean grinder and stored at room temperature until required.

### 2.2. Fucoidan Extraction

The seaweed was defatted by extracting lipids and pigments using a mixture of methanol, dichloromethane and water in a ratio of 4:2:1, as described previously [20,21]. Fucoidan was extracted from *E. radiata* using the hot water extraction method [22] with minor modifications. Briefly, 15 g of dry defatted seaweed powder was suspended in 450 mL of distilled water at a mass loading of 1:30. The mixture was heated to 70 °C with agitation overnight. The extracted fucoidan yield was expressed as a percentage of the dry seaweed weight (% dry wt).

### 2.3. Structural Analysis of Extracted Fucoidan

#### 2.3.1. Fourier Transform Infrared Spectrometer (FT-IR) Analysis

About 100 mg of ground fucoidan was analysed by Fourier transform infrared spectroscopy (FTIR) using a Model 100 FT-IR spectrometer system (PerkinElmer^®^, Waltham, MA, USA). The signals were automatically recorded by averaging four scans over the range of 4000–650 cm^−1^. The Spectrum One software (version 1.2.1) was used to carry out the baseline and ATR corrections for penetration depth and frequency variations.

#### 2.3.2. NMR Spectroscopy Analysis

The *E. radiata* fucoidan extract (10 mg/mL) was dissolved in 1 mL of D_2_O, followed by centrifugation at 13,000× *g* for 2 min. The supernatant was filtered through a 0.45-µm filter to remove any insoluble material. The deuterium-exchanged sample was subjected to ^1^H-NMR analysis. Spectra were recorded at 23 °C using a 400 MHz spectrometer (Bruker, Fällanden, Switzerland) equipped with Topspin 3.5 software (Bruker, Billerica, MA, USA). The chemical shifts were expressed in ppm.

#### 2.3.3. Thermogravimetric Analysis 

Thermogravimetric analysis of *E. radiata* fucoidan was conducted with a Pyris Diamond model thermogravimetric analyser (PerkinElmer^®^, Shelton, CT, USA). Approximately 4 mg of fucoidan was placed in an alumina crucible. Pure nitrogen (purity of 99.99%), with a 20 mL/min flow rate, was used as a carrier gas to extinguish the mass transfer effect to a minimum level. The fucoidan was heated from 30 °C to 900 °C at a heating rate of 30 °C/min. A separate blank run using an empty pan was conducted for baseline correction. The weight loss relative to the temperature increment was automatically recorded.

### 2.4. Chemical Composition Analysis of Fucoidan

The total sugar content of fucoidan was analysed using the phenol-sulfuric acid method, with l-fucose as a standard [23]. Partially hydrolysed fucoidan total reducing sugar content was determined according to the previously established dinitrosalicylic acid (DNS) method [24]. Protein content was measured by the Bradford method using bovine serum albumin as the standard [25]. Formic acid (60% *v/v*) was used to desulphurise the fucoidan’s sulphate content, which was measured using a modified barium chloride-gelatin method described previously [26]. The total polyphenols were determined using a modified Folin-Ciocalteu method [27]. Also, the ash content in fucoidan was derived from derivative thermogravimetry (DTG) data obtained during TGA analysis. Monosaccharides, including L-fucose, D-glucose, D-galactose, D-mannose, L-arabinose and D-fructose, generated from 2 M trifluoroacetic acid (TFA) hydrolysis of fucoidan were quantified using a Shimadzu (Kyoto, Japan) high-performance liquid chromatography (HPLC) instrument equipped with a refractive index (RID) detector. The neutral sugars were separated using a Fortis Amino analytical column (150 mm × 4.6 mm) with slight modification of the recommended method from the supplier (Fortis Technologies Ltd., Neston, UK). The mobile phase was a mixture of HPLC grade acetonitrile and degassed H_2_O (Milli-Q, MERCK) in a ratio of 3:1. The flow rate was 0.8 mL/min, and the column temperature was at 30 °C with a sample injection volume of 20 µL. Finally, uronic acids, d-galacturonic acid and d-glucuronic acid, were quantified enzymatically according to the microtiter plate format described in the Megazyme^TM^ K-URONIC kit.

### 2.5. Determination of Average Molecular Weight

The molecular weight of the extracted fucoidan was estimated using analytical ultracentrifugation. A volume of 15 mL of *E. radiata* fucoidan stock solution (0.5 mg/mL) prepared in distilled water was transferred into and filtered through 100 K, 50 K, 30 K and 10 K Amicon^®^ ultra-centrifugation filters (MERCK). The retentates were obtained by centrifuging the filters at 4000 g for 20 min. The filtrate and retentate samples from each filtration step were analysed for the presence of fucoidan according to the phenol-sulfuric acid assay.

### 2.6. Carbohydrate Digesting Enzymes Inhibition Studies

#### 2.6.1. α-Amylase Activity Assay

The inhibition potential of the extracted *E. radiata* fucoidan and the controls (*F. vesiculosus* fucoidan and acarbose) were investigated and compared using the α-amylase activity assay. In brief, 2% (*w/v*) potato starch dissolved in 0.05 M sodium phosphate buffer (pH~7.0) was mixed with variable concentrations of potential inhibitors ranging from 0.01–1 mg/mL. The reaction was started by adding porcine α-amylase (10 units/mL in a total reaction volume of 400 µL). The reaction mixture was incubated at 37 °C for 20 min with gentle agitation at 70 rpm. The amount of reducing sugars produced was measured according to the DNS method [24]. A control reaction was prepared using the same procedure replacing the inhibitor sample with distilled water. Extrapolation of absorbance values was performed against a glucose standard calibration curve. The α-amylase inhibition was expressed as a relative product (reducing sugar (RS)) percentage according to the following formula:

(1) $$Enzyme\;inhibition\;\%=\frac{\left(RS\;released\;by\;control-RS\;released\;by\;test\;reaction\right)}{\;RS\;released\;by\;control}\;\times 100$$

The inhibitor concentration resulting in 50% inhibition of α-amylase activity (IC_50_) was determined graphically using GraphPad Prism Software version 6.0 (GraphPad Inc., San Diego, CA, USA).

#### 2.6.2. α-Glucosidase Activity Assay

The inhibition of α-glucosidase activity was determined by dissolving the substrate, 10 mM *p*-nitrophenyl-α-D-glucopyranoside (*p*NPG), in 0.05 M sodium phosphate buffer (pH~7.0) in the presence of varied concentrations (0.01–1 mg/mL) of potential inhibitors. The reaction was started by adding α-glucosidase (0.1 units/mL) to a total reaction volume of 400 µL. The reaction mixture was incubated at 37 °C for 20 min and terminated by the addition of 400 µL of 2M NaCO_3_ solution. The reaction mixture with water instead of the inhibitors was used as a negative control. The absorbance of the released *p*-nitrophenol was measured at 405 nm. The amount of *p*-nitrophenol produced was extrapolated from a *p*-nitrophenol standard curve. The α-glucosidase % inhibition was calculated as relative product (*p*-nitrophenol (*p*N)) percentage as follows:

(2) $$Enzyme\;inhibition\;\%=\frac{\left(pN\;released\;by\;control-pN\;\;released\;by\;test\;reaction\right)}{pN\;\;released\;by\;control}\;\times 100$$

The inhibitor concentrations resulting in 50% inhibition of enzyme activity (IC_50_) was determined graphically using the GraphPad Prism Software version 6.0 (GraphPad Inc., San Diego, CA, USA).

### 2.7. Interaction between Fucoidan and α-Glucosidase

#### 2.7.1. Tryptophan Fluorescence Analysis of the α-Glucosidase-Fucoidan Interaction 

The α-glucosidase-fucoidan interaction was analysed through the intrinsic tryptophan fluorescence of the enzyme [28]. Briefly, 20 µg/mL of α-glucosidase and *E. radiata* fucoidan (0.0625–0.5 mg/mL) in 0.05 M sodium phosphate buffer (pH 7.4) were incubated for 20 min at 37 °C. Thereafter, fluorescence was measured between 300 and 500 nm after initial excitation at 295 nm using a SpectraMax M3 (Separations, Roodeport, South Africa) microplate reader at 25 °C using standard 96-well black microplates with 5 nm increments. The relative fluorescence was calculated as the average value obtained from at least 4 spectrum scans corrected for their baseline (buffer with or without inhibitors in the absence of enzyme) reading.

#### 2.7.2. Determination of Binding Parameters of the α-Glucosidase-Fucoidan Interaction

To further elucidate the quenching mechanism of *E. radiata* fucoidan, the fluorescence data was analysed by constructing a modified Stern-Volmer plot ((F_0_/F_0_-F) versus 1/[fucoidan]), where F_0_ and F are the fluorescence intensities of α-glucosidase in the absence and presence of the quencher (fucoidan), respectively. The values for the association constant (K) and the number of binding sites (n) were obtained from the intercept and slope of the constructed secondary plot, respectively.

#### 2.7.3. Circular Dichroism Analysis of Secondary Structural Changes of α-Glucosidase upon Interaction with *E. radiata* Fucoidan

The secondary structural conformation of the α-glucosidase (upon interacting with fucoidan) was analysed using Far-UV circular dichroism (CD) as previously described [29]. Briefly, 0.2 µM of α-glucosidase was suspended in 0.05 M phosphate buffer, pH 7.0. The analysis was conducted on a Chirascan v.4.4.1 Build spectrometer (Applied Photophysics Ltd., Leatherhead, UK) equipped with a Peltier temperature controller at 19 °C, using a 0.1 cm path-length quartz cuvette (Hellma Analytics, Baden-Württemberg, Germany). The data was analysed and deconvoluted to α-helix, β-sheet, β-turns and unordered regions using the CONTIN program of the Dichroweb online server (accessed 3 October 2019) [30]. The procedure was repeated on α-glucosidase, which was pre-treated with varying concentrations of fucoidan, and a boiled enzyme positive control was included in the assay.

#### 2.7.4. Mode of α-Glucosidase Inhibition 

The α-glucosidase activity was assayed in the presence of fucoidan at fixed concentrations between 0 and 1 mg/mL in the presence of varying *p*NPG concentrations (0.25–6.25 mM), according to the protocol previously described in Section 2.6.2. The enzymatic reaction rates (*v*_0_) were calculated using the *p*-nitrophenol released. A Michaelis-Menten curve was constructed, and the *V_max_* and *K*_M_ values were determined using GraphPad Prism v. 6.0 software (GraphPad Inc., San Diego, CA, USA).

### 2.8. Investigating the Synergistic Potential of Extracted Fucoidan with Acarbose 

The α-glucosidase assay was performed as described in Section 2.6.2 with varying concentrations of inhibitors from 0 to 2 mg/mL in the reaction. The combination (acarbose: fucoidan) was maintained at a constant ratio (1:1) to determine the dose-dependent effect of the inhibitors acting alone or in combination. The % inhibition was represented as normalised enzyme inhibition using GraphPad Prism v.6.0. Also, IC_25_, IC_50_ and IC_75_ were determined for each inhibitor. The combinations of these inhibitor potencies (non-constant ratios) were investigated as per the α-glucosidase assay protocol. The synergistic effect of inhibitors was calculated by using the combination index (CI). The CI, a quantification of the degree of inhibitor interactions based on the median-effect principle, was calculated as described previously by Chou and colleagues using the CompuSyn software (ComboSyn Inc., Paramus, NJ, USA) [18].

The median-effect equation is stated below:

(3) $$log\left(\frac{fa}{fu}\right)=mlogD-mlogD_{m}$$

where *fa* is the fraction affected by dose *D*, *fu* is the unaffected fraction *(fu = 1 − fa)*, *m* is the coefficient signifying the shape of the dose-effect curve, *D* is the inhibitor and *D_m_* is the median-effect dose (IC_50_ in this case).

The *CI* was determined through the equation below:

(4) $$CI=\frac{D_{1}}{{\left(D_{x}\right)}_{1}}+\frac{D_{2}}{{\left(D_{x}\right)}_{2}}$$

where *D_1_* and *D_2_* are the doses of inhibitors that produce a certain level of inhibition in the combination system and *(D_x_)_1_* and *(D_x_)_2_* are the doses of inhibitors added alone that lead to the same level of inhibition. CI was calculated from the data as a measure of the interaction among drugs. CI values lower than 1 indicate synergy, CI values equal to 1 indicate an additive effect and CI values higher than 1 indicate antagonism.

### 2.9. Fucoidan Toxicity Screening

#### 2.9.1. Cell Culture

The HCT116 human colon cancer cell line was purchased from the American Type Culture Collection (ATCC CCL-247). The cell line was cultured in Dulbecco’s Modified Eagle’s Media (DMEM) with GlutaMAX™-I, 10% (*v/v*) FBS and 1% (*v/v*) sodium pyruvate and was maintained at 37 °C with 9% CO_2_ in a humidified atmosphere.

#### 2.9.2. Resazurin Assay

The extracted fucoidan was screened for its potential cytotoxicity on the HCT116 colon cell line using the resazurin assay. Cells were seeded at density of 1 × 10^5^ cells/well in DMEM growth media in a 96 well plate. The cells were allowed to adhere to the plate matrix overnight at 37 °C in 9% CO_2_ and then treated with fucoidan extract in the range 0.1 mg/mL to 2.5 mg/mL in the reaction. Also, fluorouracil (5-FU) in the concentration range of 0.0064 µM to 2500 µM was included as a positive control for cytotoxicity. The treated and untreated cultures were incubated for another 72 h. Thereafter, resazurin (0.54 mM) was added to each well and further incubated for 3 h. After the incubation, fluorescence was measured (excitation = 560 nm and emission = 590 nm). The experiment was done in biological triplicates. The half maximum response concentration (IC_50_) was determined by non-linear regression analysis using GraphPad Prism v. 6.0.

### 2.10. Statistical Analysis

The experiments were repeated in biological triplicate and the data was expressed as means ± standard deviation (SD) where applicable. One-way analysis of variance (ANOVA) determined significant differences between the enzyme activity in the absence and presence of inhibitors with 95% confidence interval where *p* < 0.05 depicted significant differences. The ANOVA tests were performed using the data analysis features in GraphPad Prism software v. 6.0. (GraphPad Inc., San Diego, CA, USA).

## 3. Results and Discussion

### 3.1. Fucoidan Yield

The *E. radiata* fucoidan was extracted using a slightly modified hot water method [31] and a yield of 5.2% (*w/w*) fucoidan was obtained from defatted seaweed. This yield was considered high as most water extraction procedures produce between 1.1 and 4.8% fucoidan [6].

### 3.2. Structural Validation of E. radiata Fucoidan

#### 3.2.1. FT-IR Spectroscopy Analysis

**Figure 1:**
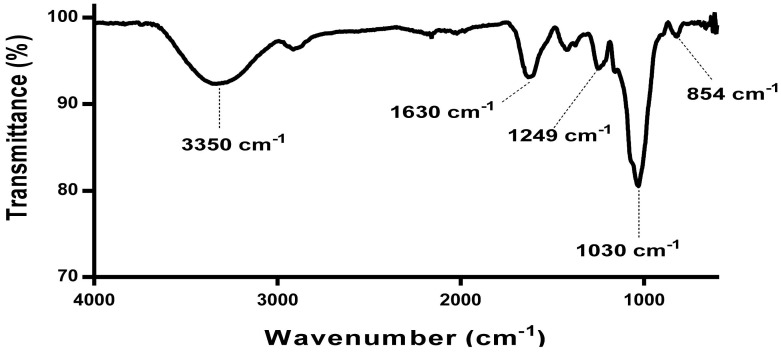
Fourier transform infrared spectra analysis of *E. radiata* fucoidan. The annotated peaks are representative of fucoidan structure reported in the literature.

The FTIR spectra for the extracted *E. radiata* fucoidan (Figure 1) was characteristic of most fucoidan extracts reported in the literature [6,19,32].

The broadened peak from wavenumber 3500 to 3000 cm^−1^ signifies the OH group stretching vibrations characteristic of most polysaccharides. The peak at 1650 cm^−1^ represents the carbonyl groups and stretching of O-acetyl groups [32]. The peaks between 1210 cm^−1^ and 1270 cm^−1^ are associated with stretching of S═O bond linked with sulphate groups [6]. Stretching vibrations of the glycosidic C─O bond are represented by the wave number close to 1100 cm^−1^ [33]. The small peak at 854 represents sulphate groups attached to the carbonyl groups of sidechains such as galactose [32]. The absence of peaks in the 1700–1715 cm^−1^ range, 808 cm^−1^ and 822 cm^−1^ (Figure 1) showed the absence or limited amounts of uronic acid contamination in the extracted fucoidan. In addition, IR bands at 940 cm^−1^ were absent, which denote stretching C─O bonds in uronic acids [34,35].

#### 3.2.2. ^1^H NMR Analysis

**Figure 2:**
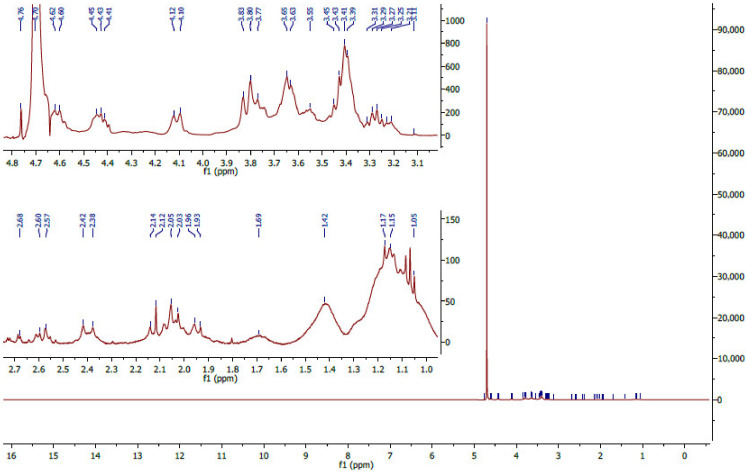
^1^H-nuclear magnetic resonance spectrum of *E. radiata* fucoidan. The spectra show peaks from 1 ppm to about 5.1 ppm, with zoom in spectra to show peaks within the regions of interest.

The structural backbone of the fucoidan extract was also validated using proton nuclear magnetic resonance. The chemical shifts in the *E. radiata* fucoidan spectra showed visible peaks between 1 ppm and 5.2 ppm (Figure 2), consistent with the NMR spectra of fucoidan reported in the literature. The chemical shifts observed in the range 1.1 to 1.5 ppm (Figure 1) suggest the presence of alternating α-1,3 and α-1,4 linkages of fucose residues (α-L-Fuc, α-L-Fuc (2-SO_3_^−^) and α-L-Fuc (2,3-diSO_3_^−^) [36]. Also, vibration bands at 1.45 ppm are assigned to symmetric CH_3_ deformations emanating from hydrogens on C6 of fucose [37]. The peak at 2.1 ppm assigned to the H-6 methylated protons of l-fucopyranosides [38] was present in the extract. Also, the extracted fucoidan showed characteristic peaks (Figure 2) in the range 3.5–4.5 ppm attributed to the (H2 to H5) ring protons of the l-fucopyranosides. The peaks in the ring proton region also suggest variable fucosal sulphates that are located at variable glycosidic linkages with varying monosaccharide patterns [38]. The prominent peak at 4.7 ppm signals the presence of 3-linked d-galactopyranosyl residues [39].

Moreover, definitive peaks were observed close to 3.3 ppm and 3.7 ppm in all extracts (Figure 2), suggesting the presence of hexoses, including, glucose, galactose and mannose [38]. These observations concurred with the chemical characterisation reported in this study, which generally showed variable monosaccharides within the extracted fucoidan (Table 1). Lastly, no chemical shifts in the region around 5.8 ppm were observed (Figure 2). Peaks in this region are representative of uronic acids and the presence of alginate impurities [40].

#### 3.2.3. Thermogravimetric Analysis

**Figure 3:**
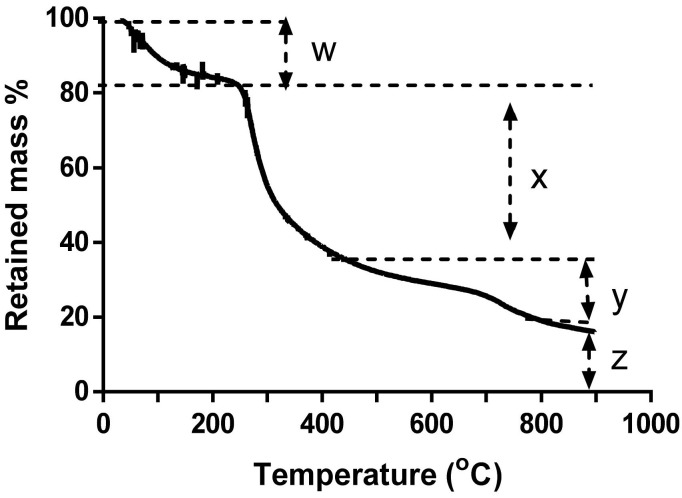
Thermogravimetric analysis (*TGA*) analysis of extracted *E. radiata* fucoidan. W—shows loss of readily volatile material (moisture content), X—represents polymer degradation, Y—combustion of carbon black and Z—ash content.

Thermogravimetric analysis of the fucoidan was done primarily to determine the ash content of fucoidan. Furthermore, fucoidan decomposition through heating validated fucoidan as a polysaccharide as its decomposition started just after 200 °C (Figure 3), characteristic of organic polymers [41].

The TGA plot of *E. radiata* fucoidan shows about 17.4% loss of mass at temperature of 240 °C (Figure 3). This decrease in mass may be due to loss in moisture content through evaporation of water [38] and some volatile matter [39]. The major loss of mass (~45%) occurred between 240 °C and 413 °C, which accounted for the arbitrary depolymerisation/decomposition of organic constituents such as carbohydrates. Above 420 °C, combustion of carbon black occurred. After heating at 900 °C, the residual mass was about 15.5% and this accounted for the ash content, which may contain sulphates, phosphates and carbonates [40].

### 3.3. Chemical Profiling and Molecular Size Estimation of E. radiata Fucoidan

*E. radiata* fucoidan was partially chemically characterised by determining total sugar content, monosaccharides and impurities, including protein, phenolics and uronic acids. Moreover, the ash content and size estimation of the fucoidan polymer is also reported in this section (Table 1). *E. radiata* fucoidan had a total carbohydrate content of 88%, of which 51% constituted total reducing sugars (Table 1). These observations showed that the fucoidan had a high carbohydrate content which concurs with findings by Daub et al. [17] who also reported a high carbohydrate content in fucoidan. The *E. radiata* fucoidan contained about 4% l-fucose and 9% sulphate. The amount of sulphate quantified in our experiments was similar to findings by Charoensiddhi and colleagues, who reported about 7% sulphate content in their extracted fucoidan [42]. HPLC was further used to determine the composition of the fucoidan upon TFA hydrolysis; it contained the monosaccharides glucose, fucose, galactose and mannose. The most prominent monosaccharide from the extracted fucoidan was glucose, one of the highest monosaccharides previously detected in fucoidan extracted from the *Ecklonia* species in the literature [19,31]. Also, the extracted fucoidan contained trace amounts of protein and phenolics (Table 1). Moreover, the extracted *E. radiata* fucoidan contained minute amounts of uronic acids (Table 1), which are the most common fucoidan impurities.

This uronic acid determination data also concurred with the NMR spectra (Figure 2), which did not identify any peaks representing uronic acids. Therefore, the extracted *E. radiata* fucoidan was deemed pure. In addition, the molecular weight of the *E. radiata* fucoidan was estimated to be >100 kDa by ultracentrifugation (Table 1). Of note, the molecular weight of *E. radiata* fucoidan extracted by Charoensiddhi et al. [42] was determined by size exclusion chromatography (SEC) to be 339.78 kDa. It is important to note that fucoidan size is an important factor in their observed biological activities, and smaller fractions are deemed better suited for bioaccessibility [43].

### 3.4. Inhibition of Carbohydrate Digestion Enzymes

#### 3.4.1. Inhibition of α-Amylase by Acarbose

**Figure 4:**
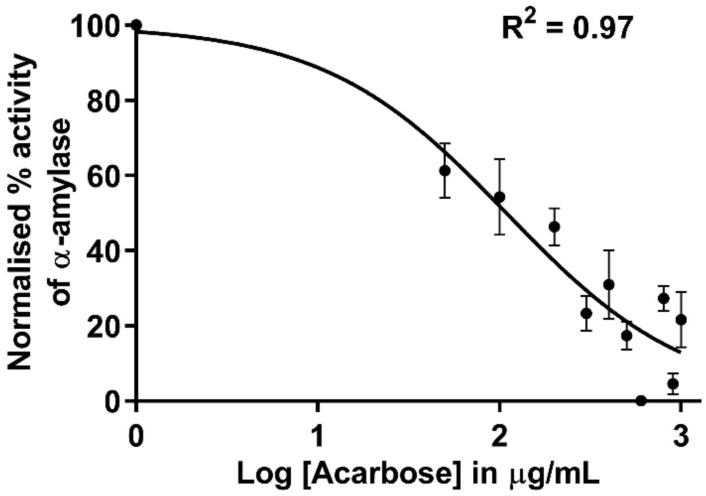
Dose- response curve of inhibition of α-amylase by acarbose. Values are represented as means ± SD (*n* = 3).

The water extracted *E. radiata* and commercial *F. vesiculosus* fucoidans did not inhibit porcine α-amylase activity (data not shown). However, the acarbose control inhibited α-amylase activity and displayed an IC_50_ of 109.1 µg/mL (Figure 4). These observations were expected for *F. vesiculosus,* as Kim and colleagues demonstrated that seaweed extracted fucoidans did not possess inhibitory activity towards α-amylase [10]. *E. radiata* fucoidan inhibition of porcine α-amylase activity has not been reported in the literature. In addition, most fucoidans reported in the literature do not display any inhibitory potential towards porcine α-amylase [17,42,43].

#### 3.4.2. Inhibition of α-Glucosidase

The extracted *E. radiata* fucoidan and commercial *F. vesiculosus* fucoidan both exhibited a significant reduction of *Saccharomyces* α-glucosidase activity—in fact, more than acarbose (Table 2). *E. radiata* fucoidan potency (IC_50_) is also comparable to that of the standard in the field, *F. vesiculosus* fucoidan (Table 2), which suggests that the extracted fucoidan is a powerful inhibitor.

Our data shows that acarbose is a weaker inhibitor of α-glucosidase compared to the fucoidan extract. Moreover, acarbose is currently used as a medication for type 2 diabetes and has well-documented side effects, including flatulence, meteorism, abdominal distension and even diarrhoea [44]. Therefore, the extracted fucoidan may constitute credible alternate sources of potent α-glucosidase inhibitors, presenting fewer side effects as natural bioproducts. Furthermore, literature has shown that most fucoidan extracts are more active in suppressing α-glucosidase activity than α-amylase. Moreover, natural product research contributing to diabetes treatment and prevention has focused more on α-glucosidase inhibition. The enzyme is directly responsible for releasing glucose from maltose and sucrose [10]. Also, the extensive inhibition of α-amylase is not desirable. Undigested carbohydrates may reach the colon, where they ferment due to bacterial degradation, which often results in diarrhoea, abdominal distension and flatulence. Therefore, our extract is a viable candidate as it inhibits α-glucosidase, but not α-amylase.

#### 3.4.3. Fucoidan Directly Interacts with α-Glucosidase

**Figure 5:**
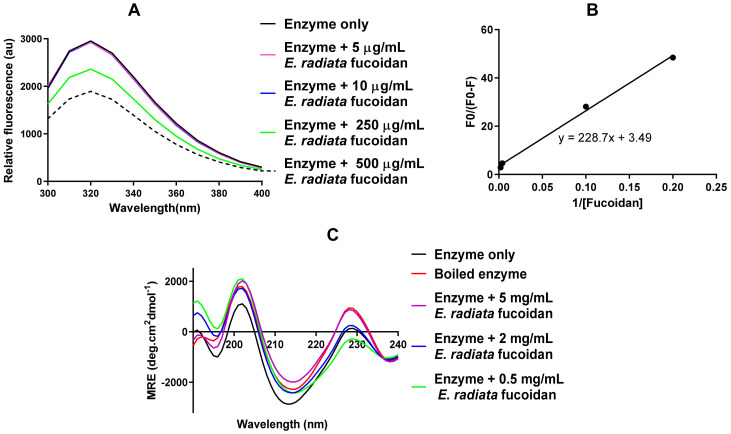
*E. radiata* fucoidan directly interacts with the α-glucosidase enzyme. (**A**) Fluorescence emission spectra of intrinsic fluorescent quenching of α-glucosidase in the presence of *E. radiata* fucoidan. (**B**) F0/(F0−F) plot versus 1/[fucoidan] for the fucoidan-α-glucosidase complex. (**C**) Circular dichroism spectra showing changes in α-glucosidase econdary structure in the presence of *E. radiata* fucoidan. Values are represented as means ± SD (*n* = 3).

The fucoidan perturbations caused conformational changes in the tertiary structure of α-glucosidase shown by the dose-dependent shifts of relative tryptophan fluorescence of the enzyme with an increase in fucoidan concentration (Figure 5A).

To consolidate the direct interaction of fucoidan and the enzyme shown in Figure 5A, a modified Stren Volmer plot was constructed (Figure 5B). From this plot, fucoidan had an *n* value of approximately 3.5, indicating that fucoidan has 3 to 4 binding sites to facilitate the interaction with α-glucosidase. Also, the binding constant (*K*) between fucoidan and α-glucosidase was calculated to be 227.8 µg/mL.

The deconvoluted α glucosidase enzyme showed the β sheet conformation predominantly with 39.6% β strands, 18.9% β turns, 38% unordered and 3.6% α helices. A similar pattern was observed within the secondary structural conformation where the presence of fucoidan shifted the deconvoluted spectrum towards the positive horizontal axis upon the addition of fucoidan (Figure 5C). This observed pattern also consolidated that fucoidan directly interacts with the α glucosidase.

These experiments suggest the direct interaction between the fucoidan and α-glucosidase, concurring with the findings by Daub and colleagues with *E. maxima* fucoidan [17]. Our findings also agree with reports which stated small shifts in the secondary and tertiary structure of α-glucosidase, which were observed in the presence of inhibitors [45,46]. Molecular modelling of *S. cerevisiae* α-glucosidase identified one active site and four allosteric sites [47]. The number of binding sites identified for *E. radiata* fucoidan, suggest that the fucoidan binding possibly occurs at the active site and allosteric sites or exclusively at the allosteric sites of α-glucosidase. The 3.5 identified binding sites do not account for full binding to all allosteric sites, indicating that the large size of fucoidan likely prohibits it from binding to all available sites equally. The mode of enzyme inhibition by fucoidan was then investigated by determining kinetic parameters using Michaelis-Menten modelling.

#### 3.4.4. Mode of Inhibition of α-Glucosidase Activity by *E. radiata* Fucoidan 

**Figure 6:**
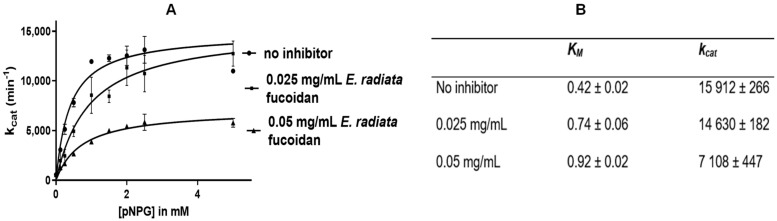
Kinetic parameters for α-glucosidase inhibition. (**A**) Michaelis Menten curve for *E. radiata* fucoidan and (**B**) Kinetic parameters to determine the mode of inhibition of *E. radiata* fucoidan on α-glucosidase. Values are represented as means ± SD (*n* =3).

*E. radiata* fucoidan was confirmed to inhibit the function of α-glucosidase (Figure 6A) and to be a mixed type inhibitor (Figure 6B).

The kinetic parameters of α-glucosidase determined from Michaelis-Menten modelling (GraphPad Prism v. 6.0) illustrated an increase in the *K_M_* and a decrease in the *kcat* with increasing inhibitor concentration (Figure 6B) which suggested fucoidan is a mixed inhibitor. Mixed inhibitors increase the *K_M_* and decrease the *V_max_* of enzymes [48]. Most fucoidans lack efficacy against α-amylase, although they are excellent inhibitors of α glucosidase. However, the vice versa is true for acarbose. The use of both compounds could prove helpful in the management of T2DM.

### 3.5. A Poly Compound (Acarbose and Fucoidan) Combination Approach in Inhibiting α-Glucosidase

**Figure 7:**
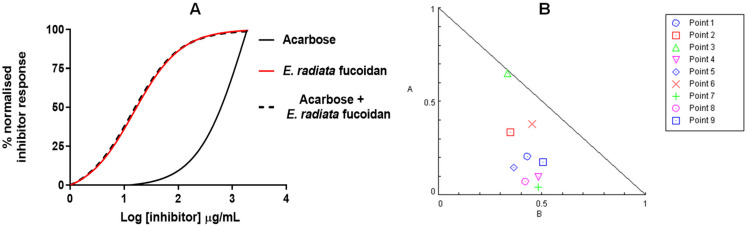
*E. radiata* fucoidan synergistically inhibits the activity of α-glucosidase. (**A**) Dose-response curves of the inhibition potential of compounds on α-glucosidase. (**B**)Normalised isobologram analysis of acarbose and *E. radiata* fucoidan combinations. The axes scales (A-acarbose & B-*E.radiata*) represents the affected fraction (inhibited enzyme). Various combinations of acarbose and *E. radiata* fucoidan based on IC_75_, IC_50_ and IC_25_ values were tested by the Compusyn software and the combination indexes determined. The points below the hypotenuse line of the triangles indicate synergy at a particular effect.

The combination of drugs may have three possible effects: additive or synergistic and/or undesirably antagonistic interaction. The prospect of the polydrug approach was investigated between the extracted fucoidan and acarbose on α-glucosidase. As fucoidan lacked efficacy in inhibiting α-amylase, quantitative synergy by the combination did not suit our intended Compusyn model. Therefore, the drug potencies of the individual compounds and acarbose-fucoidan combinations (at constant ratios) on α-glucosidase were investigated. As shown in Figure 7A, it was evident that fucoidan is a better drug than acarbose. The acarbose-fucoidan combination showed some additive to synergistic effects (line slightly shifted to the left) at some concentration points. The Chou and Talalay method was used to investigate further and quantify the potential synergistic interactions of these compounds [49]. Briefly, the experimental data for the α-glucosidase assay were analysed using Compusyn software. Thereafter, normalised isobolograms were generated using Compusyn software [49]. The isobolograms visually illustrate synergistic combinations (Figure 7B).

**Figure 8:**
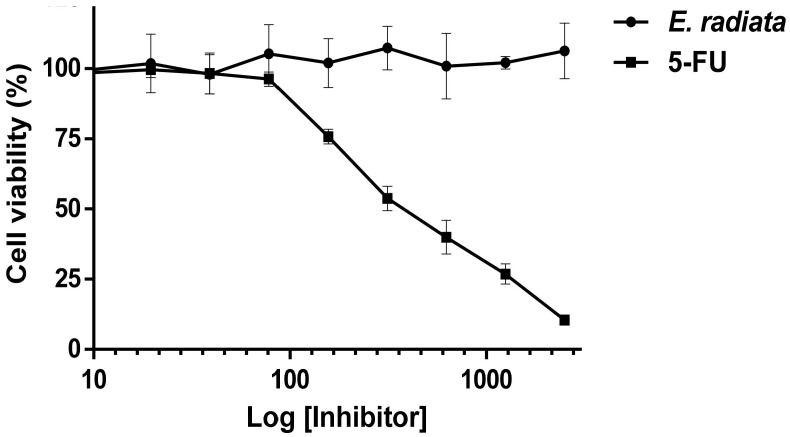
Cytotoxic effects of *E. radiata* fucoidan and 5-fluorouracil (5-FU) on HCT116 cancer cells. Cell viability was assessed by the resazurin assay. The 5-FU concentration is represented as logarithmic concentrations in µM and the *E. radiata* fucoidan as logarithmic concentrations µg/mL. Points represent means of biological replicates (*n* = 3). The error bars shown represent the standard deviations about the mean.

All the points below the hypotenuse show synergistic points; those on the line illustrate additivity, and antagonistic combinations are plotted above the line (Figure 8). The further the points are from the line, the stronger the effect, whether synergistic or antagonistic. The best synergistic combinations for the two compounds were at IC_25_:IC_50_, IC_50_:IC_50_ and IC_25_:IC_75_ [Acarbose]: [*E. radiata*] with total inhibitions at 70.4%, 74.2% and 79.4%, respectively (Table 3).

These data suggest that combinations of extracted fucoidan with acarbose act synergistically, giving rise to a potential combination therapy for T2DM. The current study has shown that fucoidan lacks efficacy towards α-amylase but is an excellent inhibitor of α-glucosidase. However, acarbose is a good inhibitor of α-amylase (Figure 4) but a less potent inhibitor of α-glucosidase (Table 2). Moreover, their combination illustrated additive or potentially synergistic interactions on α glucosidase (Figure 7 and Table 3). The synergistic potential of fucoidan has been investigated using this approach to suppress the proliferation of the measles virus [15]. More studies may be necessary to investigate fucoidan application within the prospects of polydrug combination approaches for the various bioactivities it possesses. Moreover, the Chou-Talalay method of quantifying synergy is the most used model [17]. Also, considering that fucoidan is a mixed inhibitor, the Chou-Talalay method is best suited to our situation, compared to Bliss models, which quantify synergy among compounds with a different mode of action, and Lewis models quantify synergy of compounds with a similar mode of action [17].

The side effects of acarbose use may be limited at its reduced dose when combined with fucoidan. In addition, a reduction in toxicity and side effects is one of the rationales of using drug combinations [16]. Also, combining compounds with non-overlapping side effects, fucoidan and acarbose, may allow for a more significant total efficacy with possibly fewer side effects. Lastly, considering fucoidan’s viscosity, the drug dose may be available for longer before excretion as high viscosity slows gastric emptying.

In summary, the extracted fucoidan showed synergistic interactions in combination with acarbose. This may be important for combination therapy prospects in regulating the activities of carbohydrate digesting enzymes in the quest to manage T2DM.

### 3.6. Cytotoxicity of Fucoidan

A cytotoxicity assay was performed on HCT116 (a human colon cancer cell line) to evaluate the safety of the extracted fucoidan. The resazurin reduction assay was applied to determine the viability of HCT116 cells treated with *E. radiata* fucoidan in the range of 5 to 2500 µg/mL for 72 h. Fucoidan did not have any cytotoxic effect on the HCT116 colon cells (Figure 8). On the other hand, 5-fluorouracil (5-FU), a known chemotherapeutic drug, was used as a positive control for cytotoxicity and demonstrated this effect on HTC116 cells with an IC_50_ of 9.9 µM (r^2^ = 0.98). The HCT116 is a relevant model for this study due to the oral administration of these compounds that may remain in the digestive tract for extended periods, especially in the colon, where food takes about 36 h to pass through [50]. No studies have been reported in the cytotoxic screening of fucoidan on “normal” human colorectal cells. Also, most normal cells are derived from cancer cell lines or transformed in some way for them to be able to divide and grow in culture [51]. Based on these facts and availability, the HCT116 colorectal cancer cell line was deemed appropriate for suggesting the lack of fucoidan toxicity to the cells. However, further studies on this aspect using “normal” colon cancer cell lines may be necessary at a later stage.

## 4. Conclusions

A biologically active non-toxic fucoidan was successfully extracted from *E. radiata* seaweed. The extracted fucoidan inhibited α-glucosidase more strongly than acarbose. Therefore, this fucoidan is an attractive drug candidate for managing post-prandial hyperglycemia, which causes type 2 diabetes mellitus (T2DM). Our data suggested that acarbose can be used in combination with *E. radiata* fucoidan, as they showed synergistic associations at selected concentrations; IC_25_:IC_50_, IC_50_:IC_50_ and IC_25_:IC_75_ [Acarbose]: [*E. radiata*], with more than 70% inhibition. Furthermore, combining acarbose and fucoidan may target both carbohydrates digesting enzymes; α-amylase and α-glucosidase. This combinatorial approach would compensate fucoidan’s lack of efficacy against α-amylase when used alone. To conclude, this study highlights the prospect of a polydrug (*E. radiata* fucoidan and acarbose) combination strategy in managing T2DM.

## Figures and Tables

**Table 1:** Chemical composition of extracted *E. radiata* fucoidan.

Components	*w/w* % ± SD
Total carbohydrate ^a^	88.1 ± 7.4
Total reducing sugars ^b^	50.9 ± 10.3
Total phenolics ^c^	1.9 ± 0.4
Sulphate content ^d^	8.8 ± 1.4
Uronic acids ^e^	2.2 ± 0.7
Total protein ^f^	2.3 ± 0.89
L-fucose ^g^	3.7 ± 0.43
Glucose ^g^	7.31 ± 0.64
Galactose ^g^	4.9 ± 1.2
Mannose ^g^	4.23 ± 0.22
Arabinose ^g^	ND
Fructose ^g^	ND
Ash content ^h^	15.5
Mw (kDa) ^i^	>100

Determined by ^a^ Phenol sulphuric acid method; ^b^ DNS method; ^c^ Folin-Ciocalteu method; ^d^ Barium chloride gelatin method; ^e^ MegazymeTM uronic acid kit; ^f^ Bradford’s assay; ^g^ HPLC (RID); ^h^ TGA; ^i^ Ultracentrifugation.

**Table 2:** Table showing IC_50_ values of *E. radiata* fucoidan, *F. vesiculosus* commercial fucoidan and acarbose obtained using non-linear fit (GraphPad Prism v 6) of normalised experimental α-glucosidase response to the presence of inhibitor. Values are represented as means with a SD < 5%.

Inhibitor	IC_50_ of Fucoidan/Compound (µg/mL)
*E. radiata*	19
*F. vesiculosus* control	16
Acarbose control	332

**Table 3:** Synergistic effects of acarbose and fucoidan extracts on α-glucosidase activity.

Points on Isobologram	Compounds Combinations	Compound Concentration (µg/mL) Acarbose: *E. radiata*		% Residual α-Glucosidase Activity ± SD	CI	Effect
1	IC_75_-IC_75_	2223	35.2	18.1 ± 2.35	0.63	Synergistic
2	IC_75_-IC_50_	2223	18.6	26.1 ± 3.64	0.68	Synergistic
3	IC_75_-IC_25_	2223	9.9	39.8 ± 2.96	0.98	Synergistic
4	IC_50_-IC_75_	922	35.2	20.1 ± 2.56	0.58	Synergistic
5	IC_50_-IC_50_	922	18.6	26.8 ± 3.4	0.51	Synergistic
6	IC_50_-IC_25_	922	9.9	47.7 ± 6.39	0.83	Synergistic
7	IC_25_-IC_75_	382	35.2	20.6 ± 3.41	0.52	Synergistic
8	IC_25_-IC_50_	382	18.6	29.6 ± 3.72	0.49	Synergistic
9	IC_25_-IC_25_	382	9.9	51.4 ± 7.2	0.68	Synergistic

CI = the combination index quantifying the degree of inhibitor interactions. *E. radiata* within the table represents fucoidan extracted from *E. radiata* seaweeds.

## Data Availability

Data available upon request.

## References

[1] WHO World Health Organization. https://www.who.int/news-room/fact-sheets/detail/diabetes.

[2] Moini J. (2019). The Epidemic and Prevalence of Diabetes in the United States. Epidemiology of Diabetes.

[3] Galicia-Garcia U., Benito-Vicente A., Jebari S., Larrea-Sebal A., Siddiqi H., Uribe K.B., Ostolaza H., Martín C. (2020). Pathophysiology of Type 2 Diabetes Mellitus. Int. J. Mol. Sci..

[4] Cho M., Han J.H., You S. (2011). Inhibitory effects of fucan sulfates on enzymatic hydrolysis of starch. LWT.

[5] Wilcox G. (2005). Insulin and Insulin Resistance. Clin. Biochem. Rev..

[6] Kumar T.V., Lakshmanasenthil S., Geetharamani D., Marudhupandi T., Suja G., Suganya P. (2015). Fucoidan. A α-d-glucosidase inhibitor from Sargassum wightii with relevance to type 2 diabetes mellitus therapy. Int. J. Biol. Macromol..

[7] Lopes G., Andrade P.B., Valentão P. (2016). Phlorotannins: Towards New Pharmacological Interventions for Diabetes Mellitus Type 2. Molecules.

[8] McIver L.A., Tripp J. StatPearls. https://www.ncbi.nlm.nih.gov/books/NBK493214/.

[9] Yuan H., Ma Q., Ye L., Piao G. (2016). The Traditional Medicine and Modern Medicine from Natural Products. Molecules.

[10] Kim K.-T., Rioux L.-E., Turgeon S.L. (2014). α-amylase and α-glucosidase inhibition is differentially modulated by fucoidan obtained from *Fucus vesiculosus* and *Ascophyllum nodosum*. Phytochemistry.

[11] Mabate B., Daub C.D., Malgas S., Edkins A.L., Pletschke B.I. (2021). Fucoidan Structure and Its Impact on Glucose Metabolism: Implications for Diabetes and Cancer Therapy. Mar. Drugs.

[12] Pozharitskaya O.N., Obluchinskaya E.D., Shikov A.N. (2020). Mechanisms of Bioactivities of Fucoidan from the Brown Seaweed *Fucus vesiculosus* L. of the Barents Sea. Mar. Drugs.

[13] Yang X.-D., Liu C.-G., Tian Y.-J., Gao D.-H., Li W.-S., Ma H.-L. (2017). Inhibitory effect of fucoidan on hypoglycemia in diabetes mellitus anim. Int J Clin Exp Med..

[14] Bolton J.J., Stegenga H. (2002). Seaweed species diversity in South Africa. South Afr. J. Mar. Sci..

[15] Morán-Santibañez K., Cruz-Suárez L.E., Ricque-Marie D., Robledo D., Freile-Pelegrin Y., Peña-Hernández M.A., Rodríguez-Padilla C., Trejo-Avila L.M. (2016). Synergistic Effects of Sulfated Polysaccharides from Mexican Seaweeds against Measles Virus. BioMed Res. Int..

[16] Greco W.R., Faessel H., Levasseur L. (1996). The Search for Cytotoxic Synergy Between Anticancer Agents: A Case of Dorothy and the Ruby Slippers?. J. Natl. Cancer Inst..

[17] Roell K.R., Reif D., Motsinger-Reif A.A. (2017). An Introduction to Terminology and Methodology of Chemical Synergy—Perspectives from Across Disciplines. Front. Pharmacol..

[18] Chou T.T.C. Compusyn. http://www.Combosyn.com.

[19] Daub C.D., Mabate B., Malgas S., Pletschke B.I. (2020). Fucoidan from *Ecklonia maxima* is a powerful inhibitor of the diabetes-related enzyme, α-glucosidase. Int. J. Biol. Macromol..

[20] Suresh V., Senthilkumar N., Thangam R., Rajkumar M., Anbazhagan C., Rengasamy R., Gunasekaran P., Kannan S., Palani P. (2013). Separation, purification and preliminary characterization of sulfated polysaccharides from *Sargassum plagiophyllum* and its in vitro anticancer and antioxidant activity. Process. Biochem..

[21] Yuan Y., Macquarrie D. (2015). Microwave assisted extraction of sulfated polysaccharides (fucoidan) from *Ascophyllum nodosum* and its antioxidant activity. Carbohydr. Polym..

[22] Lee S.-H., Ko C.-I., Ahn G., You S., Kim J.-S., Heu M.S., Kim J., Jee Y., Jeon Y.-J. (2012). Molecular characteristics and anti-inflammatory activity of the fucoidan extracted from *Ecklonia cava*. Carbohydr. Polym..

[23] Dubois M., Gilles K.A., Hamilton J.K., Rebers P.A., Smith F. (1956). Colorimetric Method for Determination of Sugars and Related Substances. Anal. Chem..

[24] Miller G.L. (1959). Use of Dinitrosalicylic Acid Reagent for Determination of Reducing Sugars. Anal. Chem..

[25] Bradford M.M. (1976). A rapid and sensitive method for the quantitation of microgram quantities of protein utilising the principle of protein dye binding. Anal. Biochem..

[26] Dodgson K.S., Price R. (1962). A note on the determination of the ester sulphate content of sulphated polysaccharides. Biochem. J..

[27] Huang D., Ou B., Prior R.L. (2005). The Chemistry behind Antioxidant Capacity Assays. J. Agric. Food Chem..

[28] Mabate B., Zininga T., Ramatsui L., Makumire S., Achilonu I., Dirr H., Shonhai A. (2018). Structural and biochemical characterization of *Plasmodium falciparum* Hsp70-x reveals functional versatility of its C-terminal EEVN motif. Proteins: Struct. Funct. Bioinform..

[29] Zininga T., Achilonu I., Hoppe H., Prinsloo E., Dirr H., Shonhai A. (2015). Overexpression, Purification and Characterisation of the *Plasmodium falciparum* Hsp70-z (PfHsp70-z) Protein. PLoS ONE.

[30] Whitmore L., Wallace B.A. (2007). Protein secondary structure analyses from circular dichroism spectroscopy: Methods and reference databases. Biopolymers.

[31] January G., Naidoo R., Kirby-McCullough B., Bauer R. (2019). Assessing methodologies for fucoidan extraction from South African brown algae. Algal Res..

[32] Fernando S., Sanjeewa K.K.A., Samarakoon K.W., Lee W.W., Kim H.-S., Kim E.-A., Gunasekara U.K.D.S.S., Abeytunga D.T.U., Nanayakkara C., De Silva E.D. (2017). FTIR characterization and antioxidant activity of water soluble crude polysaccharides of Sri Lankan marine algae. ALGAE.

[33] Pereira L., Gheda S.F., Ribeiro-Claro P.J.A. (2013). Analysis by Vibrational Spectroscopy of Seaweed Polysaccharides with Potential Use in Food, Pharmaceutical, and Cosmetic Industries. Int. J. Carbohydr. Chem..

[34] Chandía N.P., Matsuhiro B., Mejías E., Moenne A. (2004). Alginic acids in *Lessonia vadosa*: Partial hydrolysis and elicitor properties of the polymannuronic acid fraction. Environ. Boil. Fishes.

[35] Leal D., Matsuhiro B., Rossi M., Caruso F. (2008). FT-IR spectra of alginic acid block fractions in three species of brown seaweeds. Carbohydr. Res..

[36] Shan X., Liu X., Hao J., Cai C., Fan F., Dun Y., Zhao X., Liu X., Li C., Yu G. (2016). In vitro and in vivo hypoglycemic effects of brown algal fucoidans. Int. J. Biol. Macromol..

[37] Kopplin G., Rokstad A.M., Mélida H., Bulone V., Skjåk-Bræk G., Aachmann F.L. (2018). Structural Characterization of Fucoidan from *Laminaria hyperborea*: Assessment of Coagulation and Inflammatory Properties and Their Structure–Function Relationship. ACS Appl. Bio Mater..

[38] Alwarsamy M., Gooneratne R., Ravichandran R. (2016). Effect of fucoidan from *Turbinaria conoides* on human lung adenocarcinoma epithelial (A549) cells. Carbohydr. Polym..

[39] Thangapandi M., Kumar A.T.T. (2013). Effect of fucoidan from *Turbinaria ornata* against marine ornamental fish pathogens. J. Coast. Life Med..

[40] Nguyen T.T., Mikkelsen M.D., Tran V.H.N., Trang V.T.D., Rhein-Knudsen N., Holck J., Rasin A.B., Cao H.T.T., Van T.T.T., Meyer A.S. (2020). Enzyme-Assisted Fucoidan Extraction from Brown Macroalgae *Fucus distichus* subsp. evanescens and *Saccharina latissima*. Mar. Drugs.

[41] Liu X., Yu W. (2006). Evaluating the thermal stability of high performance fibers by TGA. J. Appl. Polym. Sci..

[42] Charoensiddhi S., Conlon M.A., Methacanon P., Franco C., Su P., Zhang W. (2017). Gut health benefits of brown seaweed *Ecklonia radiata* and its polysaccharides demonstrated in vivo in a rat model. J. Funct. Foods.

[43] Fitton J.H., Stringer D.N., Karpiniec S.S. (2015). Therapies from Fucoidan: An Update. Mar. Drugs.

[44] Kotowaroo M.I., Mahomoodally M.F., Gurib-Fakim A., Subratty A.H. (2006). Screening of Traditional Anti-diabetic Medicinal Plants of Mauritius for Possible α-Amylase Inhibitory Effects in vitro. Phytother.Res..

[45] Liu M., Zhang W., Wei J., Lin X. (2011). Synthesis and α-Glucosidase Inhibitory Mechanisms of Bis(2,3-dibromo-4,5-dihydroxybenzyl) Ether, a Potential Marine Bromophenol α-Glucosidase Inhibitor. Mar. Drugs.

[46] Ma H., Wang L., Niesen D.B., Cai A., Cho B.P., Tan W., Gu Q., Xu J., Seeram N.P. (2015). Structure activity related, mechanistic, and modeling studies of gallotannins containing a glucitol-core and α-glucosidase. RSC Adv..

[47] Şöhretoğlu D., Sari S., Özel A., Barut B. (2017). α-Glucosidase inhibitory effect of Potentilla astracanica and some isoflavones: Inhibition kinetics and mechanistic insights through in vitro and in silico studies. Int. J. Biol. Macromol..

[48] Lopina O.D., Senturk M. (2017). Enzyme inhibitors and activators. Enzyme Inhibitors and Activators.

[49] Chou T.C. (2010). Drug Combination Studies and Their Synergy Quantification Using the Chou-Talalay Method. Cancer Res..

[50] Goldman L., Bennett J.C., Goldman L., Schafer A.I. (2000). Disorders of gastrointestinal motility. Cecil Medicine.

[51] Stauffer J.S., Manzano L.A., Balch G.C., Merriman R.L., Tanzer L.R., Moyer M.P. (1995). Development and characterization of normal colonic epithelial cell lines derived from normal mucosa of patients with colon cancer. Am. J. Surg..

